# Palliative Care in Paediatric Oncology: an Update

**DOI:** 10.1007/s11912-021-01170-3

**Published:** 2022-01-21

**Authors:** Naveen Salins, Sean Hughes, Nancy Preston

**Affiliations:** 1 Department of Palliative Medicine and Supportive Care, Kasturba Medical College Manipal, Manipal Academy of Higher Education, Manipal, Karnataka 576104 India; 2 Division of Health Research, Health Innovation One, Lancaster University, Sir John Fisher Drive, Lancaster, LA1 4AT UK

**Keywords:** Cancer, Oncology, Children, Paediatric, Palliative

## Abstract

**Purpose of this Review:**

The purpose of this review is to describe the evolution of palliative care in paediatric oncology, the needs of children and their families in a paediatric oncology setting, palliative care referral practices in paediatric oncology, outcomes of palliative care referral in paediatric oncology and models of palliative care in paediatric oncology.

**Recent Findings:**

Cancer constitutes 5.2% of the palliative care needs in children. Approximately, 90% of children with cancer lives in low and middle-income countries, constituting 84% of the global burden of childhood cancers. Children in low and middle-income countries have low cure rates and high death rates making palliative care relevant in a paediatric oncology setting. Children with cancer experience pain and physical symptoms, low mood, anxiety, and fear. They feel less resilient, experience low self-worth, and have challenges coping with the illness. The families lead very stressful lives, navigating the hospital environment, and dealing with uncertainties of the future. Palliative care referral in children with cancer improves physical symptoms, emotional support, and quality of life. It enables communication between families and health care providers. It improves end-of-life care support to children and their families and facilitates less invasive diagnostic and therapeutic interventions at the end of life. Worldwide children with cancer are infrequently referred to palliative care and referred late in the illness trajectory. Most of the children referred to palliative care receive some form of cancer-directed therapy in their last days. Children in low and low-middle income countries are less likely to access palliative care due to a lack of awareness amongst paediatric oncologists about palliative care and the reduced number of services providing palliative care. A three-tier model is proposed to provide palliative care in paediatric oncology, where most children with palliative care needs are managed by paediatric oncologists and a smaller number with complex physical and psychosocial needs are managed by paediatric palliative care specialists. There are several palliative care models in paediatric oncology practised globally. However, no one model was considered better or superior, and the choice of model depended on the need, preferences identified, and available resources.

**Summary:**

Children with cancer are sparingly referred to palliative care and referred late and oncologists and haematologists gatekeep the referral process. Knowledge on palliative care referral in paediatric oncology settings might enhance collaboration between paediatric oncology and paediatric palliative care.

## Introduction

Children’s palliative care provides active, holistic care for children and young people with life-limiting illnesses [1]. It is provided from the point of a child’s diagnosis, throughout the child’s life, death, and bereavement. It encompasses all elements of quality of life; physical, emotional, social, and spiritual. It focuses on enhancing a child’s quality of life and providing support to the family. The scope of palliative care provision is not limited to symptom management, but includes respite services, end of life care, and bereavement support [1]. Palliative care is provided regardless of whether or not a child receives treatment directed at the disease and should be incorporated alongside active disease-directed therapies [2]. Multidisciplinary palliative care support is individualised according to the child’s needs. The extent of support from the palliative care team might vary depending upon the child’s illness and response to therapy [1]. It is essential to provide children’s palliative care in tertiary, secondary hospitals and the community to ensure adequate and uninterrupted care [2].

Compared to the 1998 World Health Organisation (WHO) definition of paediatric palliative care [2], the 2013 definition provided an augmented scope [1]. It included aspects relating to spiritual elements of care and enhancing the quality of life [3]. Furthermore, it also incorporated respite care, bereavement support and personalising the care approach [3]. Some oncologists felt that the paediatric palliative care definition is unclear and without distinct boundaries due to the overlap in care components between two disciplines [4]. Although most oncologists comprehended the modern definition of palliative care, integrating palliative care into paediatric oncology practice was often less well understood [5•].

Globally, cancer constitutes only 5.2% of the palliative care needs in children [6]. The lower percentage is attributed to the uncommonness of childhood cancers and the success of currently available treatment [7]. Excellent outcomes in childhood cancer have diminished palliative care’s relevance in paediatric oncology [8, 9]. However, palliative care continues to be identified with oncology where it originated and inequitable referral of children with non-cancer conditions to palliative care services [10]. It is estimated that 90% of children with cancer live in low and middle-income countries, constituting 84% of the global burden of childhood cancers [11]. Moreover, palliative care in paediatric oncology remains relevant in low and low-middle income countries due to low cure rates and high death rates [12, 13]. In this review, we discuss the evolution of palliative care in paediatric oncology, the needs of children and their families in a paediatric oncology setting, palliative care referral practices in paediatric oncology, outcomes of palliative care referral in paediatric oncology, and models of palliative care in paediatric oncology.

## Evolution of Palliative Care in Paediatric Oncology

In 1997, The Association for Children’s Palliative Care (ACT) and the Royal College of Paediatrics and Child Health (RCPCH) jointly categorised chronic and life-limiting conditions [14, 15]. The categorisation of conditions is provided in Table 1. Childhood cancers were categorised as ACT 1 [14]. All these conditions were suitable for palliative care, but the nature and level of palliative care involvement varied significantly from one category to another [15].

**Table 1:** Categorisation of chronic and life-limiting conditions in paediatrics

ACT category	Description	Key characteristics	Examples
1	Curative treatment may be feasible but can fail. Palliative care may be necessary during phases of prognostic uncertainty and when treatment fails	Possibility of cure	Cancer and certain cardiac conditions
2	A long duration of intensive treatment needed to prolong life, but premature death is still possible	Prolonged course with predominantly normal phase, waxing, and waning of health conditions	Cystic fibrosis, muscular dystrophy, and HIV/AIDS with antiretroviral treatment
3	Progressive conditions without curative treatment options, in which treatment is exclusively palliative	Prolonged course with progressive worsening	Batten’s disease, mucopolysaccharidosis, and HIV/AIDS without antiretroviral treatment
4	Conditions with severe neurological disability, which may cause weakness and susceptibility to health complications and may deteriorate unpredictably but are not considered progressive	Non-progressive condition with an unpredictable course	Cerebral palsy

The first guidelines on integrated palliative care in paediatric oncology were published in 2013 [16]. Guidelines mandated creating a specialist interdisciplinary team that can provide collaborative multi-modal care across all settings. The purpose was to provide support to children and their families whilst ensuring the child’s quality of life and safety. It advocated for child and family participation during communication and decision making. Moreover, guidelines emphasised the understanding of ethical aspects of care during a child’s end of life experience [16]. Another integrated palliative care model in paediatric oncology highlighted the provision of palliative care concurrently regardless of the disease status and improving palliative care familiarity by building relationships between palliative care specialists and paediatric oncologists [17]. Furthermore, palliative care was proposed as a standard of care in paediatric oncology [18], and the American Society of Clinical Oncology (ASCO) in one of its publications recommended the integration of palliative care in the routine care of children, adolescents, and young adults with cancer [19].

## Needs of Children and their Families in a Paediatric Oncology Setting

Pain is one of the common symptoms experienced by a child with cancer [20]. Pain is not just limited to the palliative phase [21–23]; children receiving active treatment also experience procedural pain whilst establishing a diagnosis and during cancer treatment [24, 25]. Pain is more often seen in children with solid tumours than haematological malignancies [26, 27]. Both children and their families have voiced inadequate pain management as a concern [28, 29], and felt that this gap needs to be addressed [30].

Breathlessness, nausea, fatigue, and loss of appetite are common non-pain symptoms seen in children with cancer during treatment and palliative phases [31, 32]. Children also had symptoms like fever, mouth ulcers, diarrhoea, headache, and neuropathy due to acute and long-term chemotherapy adverse effects [33]. They experienced low mood, worry, anxiety, fear, irritability, and anger [34, 35]. They also had difficulties conveying their feelings and often reported poor concentration, low energy, and lack of motivation [23, 32]. They felt less resilient, experienced low self-worth, and had challenges coping with illness [36]. They also reported body image issues that caught their peers’ attention, and they experienced peer isolation and bullying [37]. Prolonged hospitalisation meant disruption to school and play and a transformation of the home-school-play cycle to the home-hospital cycle, which children found very distressing [38].

Children with cancer were often not informed about their diagnosis by their health care providers, and the older children, who were able to comprehend, felt a need to know [39, 40]. Adolescents with cancer felt that the illness destroyed the hope of a promising future [41]. Moreover, fear of death amongst children was seldom addressed [42]. Although some could reconcile and find meaning in life situations, others were not at peace and had a sense of loss [43].

The parents of children with cancer found it challenging to manage emotional issues like feelings of isolation and behavioural changes of their children [44]. The families led very stressful lives, navigating the hospital environment and dealing with uncertainties of the future whilst ensuring timely treatment of their child [36, 45, 46]. Families also needed financial help, practical tips on managing the child at home, and supporting other children [47, 48]. Moreover, financial difficulties contributed to psychological distress amongst parents [49].

Families felt that continuous communication between parents, family caregivers, and health care providers is crucial during a child’s end of life care to facilitate decision-making [50–52] and integrated palliative care [53•]. Learning about a child’s diagnosis was highly distressing for parents [54•]. Commonly, they did not receive clear and honest communication about their child’s illness, and they emphasised the need to know about their child’s prognosis and what to expect [51, 55]. Families often appreciated sensitive communication that preserved hope rather than blunt truth-telling [56]. There was often a gap between the child’s understanding and experience of illness and parental perception of what the child knows [57•]. This gap led to barriers in communication within families.

In a terminal care setting, families often felt unprepared to deal with their child’s death [52, 58]. They were either surprised by the child dying quickly or anxious about the long wait for the child’s death [59]. They found it difficult to let go of the child’s cancer treatment, and transition to palliative care and adaptation to the new care provider was challenging [60, 61]. Families perceived involving palliative care as letting go of their child’s treatment and experienced feelings of abandonment [58, 61, 62].

In hindsight, some families regretted providing acute hospital-based care for their child during the terminal phase of illness and regretted their child receiving cancer-directed therapy at the end of life [50, 63]. Most parents experienced anticipatory grief and guilt, and poorly controlled symptoms at the child’s end of life was a common trigger for complex bereavement amongst the parents [50, 64–66]. Furthermore, parents found grief isolating due to a lack of societal understanding of their grief [67]. Although all studies supporting the views on society and grief in the narrative review were from North America [67], similar observations were made in an Indian study where grief associated with perinatal loss and a child’s death was inadequately recognised by the families, healthcare providers, and the society [68].

## Palliative Care Referral Practices in Paediatric Oncology

Globally, children with cancer were infrequently referred to palliative care and referred late in the illness trajectory [69–74]. The median time gap from cancer diagnosis to palliative care referral was eighteen months [75]. Only 16% was referred at the initial diagnosis, and 58% of the referrals happened after a cancer relapse [70]. Moreover, in most studies, palliative care referral occurred only in the last days of their lives [31, 70, 72, 76]. In a recent systematic review, internationally, the median duration between specialist palliative care referral and death was 19 days [77•]. However, this was not a paediatric specific review.

Similar to adult cancer setting, children with haematological malignancies were referred to palliative care less often than children with solid tumours [31, 78–81] and opportunities for palliative care referral and integration in these settings were often missed [82•]. Many children referred to palliative care received some form of chemotherapy in their last days [31, 72, 73, 80]. Amongst the children referred, reasons underpinning the referral, goals of care discussions, and the decision-making process for referral were seldom documented [71, 78, 83]. Presence of symptoms, psychosocial needs, and poor prognosis commonly activated a palliative care referral [84••, 85••]. However, not addressing uncertainties and avoiding conversations about death and dying often hindered referral in a paediatric oncology setting [86••]. Non-referral and delayed referral often led to invasive medical interventions at the end of life [73, 79] and increased in-hospital deaths [72, 73, 79, 87]. There was a mixed view of trial participation and palliative care referral with enrolment in one clinical trial resulting in deferred palliative care referral [88] but in another study in a clinical trial setting this did not happen [89].

Children in low and low-middle income countries are less likely to access palliative care due to a lack of awareness amongst paediatric oncologists about palliative care and the reduced number of services providing palliative care [90•]. Two Indian studies showed that 86% of children with cancer received chemotherapy during the last month of their life, and 78% was referred after cancer-directed therapy was completed, which hindered palliative care access [91, 92]. These studies mirrored palliative care referral practices in the Indian adult cancer settings [93–97]. Non-referral and late referral to palliative care in paediatric oncology in Malaysia, Nigeria, and Morocco led to poorly controlled symptoms, caregiver distress, and most of the children dying within a few weeks of palliative care referral [35, 98–100]. Moreover, availability and access to paediatric palliative care in these settings were proportional to the country’s health spending [101, 102•, 103••]. Low and low-middle income countries had limited access to opioids, lack of interdisciplinary care, and families were less empowered to participate in decision-making [101, 103••]. Lack of palliative care funding, advocacy and leadership were the other factors hindering the capacity to provide paediatric palliative care [104–106]. Similar observations were made in a narrative review that identified lack of opioids and fear of opioids, lack of specialist paediatric palliative care education, lack of a national and institutional palliative care policy, lack of awareness about palliative care needs, and lack of integration of palliative care in health systems as the key barriers hindering palliative care development in low and low-middle income countries [107]. The Paediatric palliative screening scale (PaPaS) was found to be an effective screening tool to assess palliative care needs of children and their families and the PaPaS score enabled clinicians to initiate timely referrals [108].

## Outcomes of Palliative Care Referral in Paediatric Oncology

Although improvement in children and their families’ quality of life is the desired outcome, there are no ideal measures that accurately reflect these outcomes of palliative care referral in paediatric oncology [109]. Improvements in the domains of quality of life have to be contextualised to a specific illness and patient population [109]. Betterment of the child’s quality of life is an essential factor motivating families to access palliative care [110]. Empirical studies have shown improvements in quality of life from palliative care referral in paediatric oncology [111–114]. However, these benefits were limited to physical and emotional domains of quality of life [113].

Symptom management is a critical component of the physical domain of quality of life. Studies have shown symptom assessment [115, 116] and symptom management benefit from paediatric palliative care referral [74, 75, 112, 115–121]. However, pain management benefit was better appreciated by children and families than the management of other symptoms [74, 75, 118, 121]. Furthermore, some oncologists felt that palliative care could enable treatment completion by managing pain and symptoms whilst the child is receiving cancer-directed therapy. Two systematic reviews substantiated the above findings [122, 123].

Referral to palliative care facilitated emotional support to children and their families [74, 118, 124–127]. Emotional support to families extended beyond the child’s death into the bereavement phase [126, 127]. Support from palliative care enabled families to have realistic hope. Moreover, children and families engaged in fun activities and experienced events that added meaning to their lives [124, 125].

Referral to palliative care in paediatric cancer settings improved communication between families and health care providers [74, 75, 119, 125–132]. Involvement of the palliative care team enabled early assessment [133]; facilitated initiation of family meetings during the child’s clinical deterioration [128] and assisted oncologists in prognostic discussions [74]. Palliative care teams participated in family communications during the discussion of end-of-life care [75, 129, 130], and helped paediatric oncologists to navigate critical communications surrounding the child’s end of life [130]. Palliative care referral enabled goals of care discussion and supported the process of shared decision-making [74, 75, 125, 131, 132, 134, 135]. Paediatric oncologists acknowledged the complexities of shared decision-making and appreciated the support they received from the palliative care team [135]. Referral facilitated documentation of the resuscitation preferences [74, 136] and the preferred place of care and death [75, 126, 137, 138]. Palliative care involvement also facilitated advance care planning and documentation of advanced directives [139, 140].

Palliative care input bettered end-of-life care support to children and their families in a paediatric cancer setting [74, 75, 111, 115, 120, 121, 124, 126, 132, 137, 139–143]. It facilitated less invasive diagnostic and therapeutic interventions at the end of life [115, 132, 137, 142], and children receiving palliative care input were less often resuscitated [74, 75]. Children had fewer elective and emergency hospital admissions [139, 141], and they had shorter hospital stays [111, 142]. There were fewer intensive care admissions [74, 121, 140] and more home deaths [70, 71, 121, 124]. Referral to palliative care reduced overall health care resource utilisation, maintained care continuity at the end of life [120, 143], and facilitated access to integrative therapies during the end of life phase [115]. However, a systematic review reported conflicting evidence on palliative care collaboration benefits on hospital admissions at the end of life and resuscitation at the time of death [144].

The families of children with cancer appreciated the healthcare system’s support beyond usual clinical management [47, 48]. They valued the support they got in terms of managing finances and assistance to manage the child’s needs at home [48]. Palliative care referral improved family satisfaction of care [114, 119, 127]. Involving a palliative care team facilitated discharge planning, hospice utilisation, and home-based care [75, 145]. Children receiving home-based care received uninterrupted care [146, 147]. The families valued the palliative care team as a reliable and cost-effective source of support [147, 148].

## Models of Palliative Care in Paediatric Oncology

In 2016, a three-tier model was proposed by Kaye and the team to provide palliative care in paediatric oncology [149], as represented in Table 2. It was developed from the earlier proposed integrative paediatric palliative oncology models [150–154]. In this approach, a vast majority of children with palliative care needs are seen by tier 1 services, whereas a smaller number with complex physical and psychosocial needs are seen by tier 3 services [149].

**Table 2:** Three-tier model of palliative care in paediatric oncology

Tier	Description
Tier 1 Specialist palliative Care	A team of specialist paediatric palliative care providers available for managing complex symptoms and psychosocial needs
Tier 2 Consultation-liaison	Presence of a consultation-liaison service, and there are triggers for palliative care referral. When the child satisfies the referral criteria, a consultation is triggered
Tier 3 Palliative approach	A palliative approach where all the oncologists, oncology trainees, and oncology nurses have basic training in palliative care

There are five palliative care models in paediatric oncology practised in various hospitals of the USA [155•] (Table 3**)**. No model was considered better or superior, and the choice of model depended on the need, preferences identified, and available resources. A centre with minimal resources may choose a trigger-based or a consultative model, whereas a centre with better resources may prefer a disease-specific embedded mode [155•]. Embedded paediatric palliative oncology models demonstrated a higher number of children accessing palliative care, longer stay with the palliative care service before death, and fewer days in the hospital during the last three months of their lives [156•, 157•]. Moreover, the embedded model facilitated better acceptance of paediatric palliative care by the paediatric oncology providers [158•], and improved palliative care education of paediatric oncology residents [159]. Furthermore, these models may be contextual to the US setting, and their transferability to other country settings, with scarce palliative care resources, might be challenging. Apart from these models, an Italian study showed that a virtual paediatric palliative care support network situated outside the hospital could partially substitute the lack of in-hospital palliative care services [160].

**Table 3:** Models of palliative care clinics in paediatric oncology

Model	Description	Site practiced
The floating clinic	A paediatric advanced care team (PACT) comprised of paediatric palliative care physician, paediatric palliative care nurse practitioners, and social workers called as a “floating unit” moves along with the child in the hospital and consults them during outpatient oncology visits, hospital admission, day-care chemotherapy, and transplantation	Boston’s Children’s Hospital, Boston
The disease-specific embedded clinic	A team of paediatric palliative care physicians and paediatric palliative care nurse practitioners are situated in a disease-specific clinic like leukaemia clinic, neuro-oncology clinic, or solid tumour clinic and they closely work with these disease-specific units and provide palliative care across all settings	St. Jude’s Children Research Hospital, Memphis
Trigger-based Clinic embedded in the oncology space	The team is comprised of paediatric palliative care physician, paediatric palliative care nurse, and social workers. A disease-specific criterion is created for palliative care referral. When the child meets the referral criteria, the oncology team initiates the referral process and seen by the paediatric palliative care team in the oncology clinic	Randy Children’s Hospital, San Diego
Consultation based clinic in the oncology space	A need-based referral initiated by oncologists to paediatric palliative care. There are no set referral criteria in this model, and the palliative care specialist is usually a dual board-certified clinician in both palliative care and oncology. The oncology team and the palliative care team see the child concurrently in the same clinic, and the child receives joint advice	Alfac Cancer and Blood Disorders centre, Atlanta
Telehealth clinic	Providing access to children and their families in a remote or rural setting beyond the geographic catchment of the regular palliative care services. The clinic is operated by a team of palliative care providers, who provide telephonic, and video-calling based consultations	Children’s Hospital and Medical Centre, Omaha

## Conclusion

In summary, paediatric palliative care is the multidisciplinary holistic care of the child and the family throughout the illness continuum. Children with cancer experience physical symptoms and reduced quality of life. Families are often unprepared to deal with the loss and find it challenging to cope with emotional issues. Palliative care referral improves quality of life, confers symptom management benefit, provides emotional support, enhances end of life care experience, and supports children and their families’ needs. Children with cancer were sparingly referred to palliative care and referred late and oncologists and haematologists gatekeep the referral process. Knowledge on palliative care referral in paediatric oncology settings has the potential to provide some strategies to enhance collaboration between paediatric oncology and paediatric palliative care.
